# Music and 25% glucose pain relief for the premature infant: a randomized clinical trial^1^

**DOI:** 10.1590/0104-1169.0029.2484

**Published:** 2014

**Authors:** Maria Vera Lúcia Moreira Leitão Cardoso, Leiliane Martins Farias, Gleicia Martins de Melo

**Affiliations:** 2 PhD, Full Professor, Departamento de Enfermagem, Universidade Federal do Ceará, Fortaleza, CE, Brazil; 3 PhD, RN, Hospital Infantil Albert Sabin, Fortaleza, CE, Brazil; 4 Master's student, Departamento de Enfermagem, Universidade Federal do Ceará, Fortaleza, CE, Brazil. Scholarship holder Coordenação de Aperfeiçoamento de Pessoal de Nível Superior (CAPES), Brazil

**Keywords:** Infant, Neonatal Nursing, Randomized Controlled Trial, Pain

## Abstract

**OBJECTIVE:**

to analyze the total Premature Infant Pain Profile scores of premature infants undergoing arterial puncture during music and 25% glucose interventions, and to assess their association with neonatal and therapeutic variables.

**METHOD:**

a randomized clinical trial with 80 premature infants; 24 in the Experimental Group 1 (music), 33 in the Experimental Group 2 (music and 25% glucose), 23 in the Positive Control Group (25% glucose). All premature infants were videotaped and a lullaby was played for ten minutes before puncture in Experimental Groups 1 and 2; 25% glucose administered in Experimental Group 2 and the Positive Control Group two minutes before puncture.

**RESULTS:**

60.0% of premature infants had moderate or maximum pain; pain scores and intervention groups were not statistically significant. Statistically significant variables: Experimental Group 1: head and chest circumference, Apgar scores, corrected gestational age; Experimental Group 2: chest circumference, Apgar scores, oxygen therapy; Positive Control group: birth weight, head circumference.

**CONCLUSION:**

neonatal variables are associated with pain in premature infants. Brazilian Registry of Clinical Trials: UTN: U1111-1123-4821.

## Introduction

For the sake of survival, newborns, particularly preterm infants (PTI) in the neonatal unit (NU) undergo numerous painful procedures, such as collection of arterial and venous blood, lumbar puncture, venipuncture and tracheal aspiration, among others^(^ 1 ^)^. 

It should be noted that PTI experience pain, but it may vary from that of the term infant because they do not have the ability to produce a strong sound, thereby they may not scream or present with a vocal response that displays a difference between discomfort and severe pain^(^ 2 ^)^.

Pain can be assessed and analyzed through parameters. Among these, there are behavioral and physiological responses, such as facial movements, crying, sleep and wake patterns, heart rate (HR), blood pressure, respiratory rate (RR), oxygen saturation (SpO_2_) and systolic blood pressure^(^ 3 ^)^. Due to the PTI's inability for verbal expression, the development of instruments to assess their pain in preterm infants became necessary. Among the scales for measurement of acute pain in preterm infants, is the *Premature Infant Pain Profile* (PIPP)^(^ 4 ^)^. The use of these instruments may favor the care of the PTI with greater safety and knowledge because it guides important aspects that accompany the reaction of the neonate to painful procedures.

When considering that there are neonatal variables (including sex, gestational age) and interventions (such as upper airway aspiration, venous and arterial punctures), which characterize and continue throughout the newborn's (NB) hospitalization, it is advocated that they can influence the pain reaction. Therefore, its assessment is extremely important in PTI. The use of non-pharmacological measures for pain relief in PTI in the NU, such as 25% glucose^(^ 5 ^)^ and music therapy^(^ 6 ^)^, are added to those.

Therefore, a question arose: what is the relationship between the neonatal and therapeutic variables and PIPP pain scores in PTI under the effect of either music, music associated with 25% glucose, or 25% glucose only? The answers to this question may direct other studies on PTI pain and enable nursing to obtain pain assessment parameters in this clientele through the PIPP and through the use of non-pharmacological measures. 

The objective of this study was to analyze total PIPP scores of PTI undergoing arterial puncture for blood collection and exposure to either music, music and 25% glucose, or 25% glucose, and whether there is an association between neonatal variables (gender, type of delivery, birth weight, corrected gestational age (GA), chronological age in days, length of stay in days, 1' and 5' Apgar, head and chest circumferences) and therapeutic variables (number of punctures, puncture site and use of oxygen therapy ) with pain scores.

## Method

This is an experimental triple-blinded analytical study, a randomized clinical trial performed at a NU of a public state hospital, located in the city of Fortaleza, Ceará, Brazil. It received funding from a larger project of the National Council for Scientific and Technological Development (CNPq), Universal Call 14/2011 n.483352/2011-0. The PTI who participated in the study received arterial puncture for examination as part of routine clinical treatment and according to the institutional protocol.

A sample size suitable to identify a difference between treatments in pain relief was estimated based on the PIPP pain scores (0-21 points) and its classification (≤ 6 for minimum pain and/or none and ≥7 to moderate pain and/or maximum pain. Thus, the parameters considered were the mean and standard deviation of the PIPP pain classification as none or minimum pain (3±4.2 points) and mean points on the scale of moderate to maximum pain (12±7.11 points). The sample size estimation considered an 80% power and a 5% significance level, using the sample size formula for experiments comparing unpaired groups. This formula is:



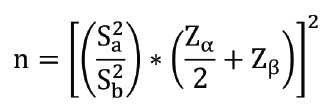



where Z_α_/Z is the value of alpha error, usually 1.96 (α=5%); Z_β_ is the value of β error, usually 0.84 (β=20%) and S_a_^2^ e S_b_^2^ are the standard deviation of the differences between groups. 

A sample size of 22 participants was calculated for each group, totaling 66 PTI. Considering the possibility of losses during the experiment, we estimated a higher total number of PTI, totaling 20% more than expected, i.e. 80 NB, 24 of these in experimental group 1 (EG1 - music); 33 in Experimental Group 2 (EG2 - music and 25% glucose); and 23 in Positive Control (PCG - 25% glucose).

Premature infants included were at a gestational age ≥32 weeks and <37 weeks; had a 5' Apgar ≥ 6; were clinically stable, with HR and RR within normal limits; received an arterial puncture for assessment; had a normal neonatal hearing screen; were on any mode of ventilation support (current oxygen, Oxi-Hood, Continuous Positive Airway Pressure or mechanical ventilation). We excluded those infants with: congenital nervous system disease, neurological malformations or impairments; medications that interfered with the nociceptive response of pain; surgical procedures; diagnoses of intraventricular hemorrhage grades III or IV; diabetic mothers; breastfeeding mothers using medications that interfered with responses to nociception. 

Collections through arterial puncture occurred from November/2011 to August/2012 during the day and night, by technical nurses on the unit. A nurse and two trained undergraduate research fellows in participated in the videotaping.

Two data collection instruments were used. The first related to neonatal variables: sex, type of birth, place of hospitalization, medical diagnosis, GA, corrected GA, chronological age (CA), birth weight, head circumference (HC), chest circumference (CC), 1 'and 5' Apgar from patients' charts; and therapeutic variables: puncture location, and amount and type of oxygen therapy, from the analysis of the video footage. The second was the PIPP pain scale, with coding based upon facial movements (brow bulge, eye squeeze and nasolabial furrow) and physiological indicators (HR and SpO_2_), at baseline (15s immediately before pain/T-15) and during pain (puncture - T30)^(^ 4 ^)^, assessing pain as none or minimum pain when scores ≤6 and as moderate to maximum when scores ≥7.

For the selection of newborns, the authors abided by the inclusion criteria. After signing of an informed consent by the infant's parents and/or guardian, a test with the musical instrument Agogo was performed in search of the sound stimulus. Whenever there was a positive auditory response, a nurse member of the research group organized sealed opaque envelopes, consecutively numbered, which were used to select the group to which the NB would be allocated. 

Two digital videorecorders were used: one directed at the face of the PTI and another directed at the monitor, at the pulse oximeter and at the Polar RS200, the latter connected to two cardiac electrodes on the thorax of the PTI to record the HR. All the PTI were fitted with headphones, but only those in EG1 and EG2 listened to a lullaby for ten minutes prior to arterial puncture, through a MP4 connected to the headphones. The newborns in EG2 and PCG were orally administered 2 ml of 25% glucose two minutes before the painful procedure through a strip of gauze soaked in the glucose solution, modified according to the size of each PTI.

Three trained nurses coded the PIPP values ​​by using the footage on an individual computer, after the reliability of observation reached a Kappa of at least 80%^(^ 7 ^)^. It is worth clarifying that each nurse received two DVDs recorded with the corresponding number of the PTI, corresponding to the face and the monitor, plus the instrument for data collection with the assessment time to be analyzed. Therefore, the three raters were blinded at all time points. 

The database was structured in an Excel spreadsheet (version 2007) for variable coding and double entry was performed to ensure data reliability. After comparing the two worksheets and correcting discrepancies, the data were exported to the Statistical Package for Social Sciences software - SPSS (version 20) to complete descriptive and comparative statistical analysis of the mean scores attributed by the three evaluators.

For the analysis of association between the categorical variables (sex and type of delivery) and the PIPP pain scores (pain and no pain), the Pearson's chi-square test or the Fisher's exact test were used, as appropriate, considering their respective assumptions. 

For comparison of the means of quantitative variables (birth weight, corrected GA, CA in days, length of stay in days, 1' and 5' Apgar, HC, CC, number of punctures) with the PIPP pain scores (pain and no pain), the Student's t-test or nonparametric Mann-Whitney test were used when the assumption of normality test could not be found. The normality of the sample data was tested using the Shapiro Wilk test. The significance level established for all tests was 5% (p <0.05).

The project was approved by the Ethics Committee in Research of the study setting, protocol 060717/11, *Plataforma Brasil* (Brazil Platform). Parents and/or guardians of the PTI signed the consent forms.

## Results

Of the 80 PTI in the sample, there was a predominance of: males in EG1, 16 (66.7%); in EG2, 18 (54.5%); and in PCG, 15 (65.2%); those born by cesarean section, 18 (75.0%) in EG1, 16 (48.5%) in EG2 and 17 (74.0%) in PCG. 

The mean birth weight was 2.179g in EG1; 2.198g in EG2; and 1.910g in the PCG. The mean 1' Apgar score in EG1, EG2 and PCG was 6.5, 6.4 and 6.7, respectively. The mean 5' Apgar increased in the groups: 8.3, 8.4 and 8.4, respectively. The mean corrected GA in weeks in the groups was 34.3% in EG1, 34.4% in EG2 and 33.7% in PCG. As for the CA in days at the time of the arterial puncture, most infants were within the first 24 hours of life in EG1 (15, 62.5%), followed by 24 hours of age in EG2 (21, 63.6%) and 48 hours of age in PCG (16, 69.5%).

At the day of data collection, most PTI were admitted to the high-risk unit: 22 (91.7%) in EG1; 23 (69.7%) in EG2; 18 (78.3%) in PCG. Respiratory Distress Syndrome + prematurity were the predominant medical diagnoses in 13 (54.2%) in SG1; 18 (54.5%) in SG2; and 13 (56.52%) in PCG.

## Dimensioning of the PIPP pain scores with neonatal and therapeutic variables

According to Table 1, it was observed that 60.0% of the PTI had moderate or maximum pain. There was no statistically significant association (p=0.398) between the pain score category and the intervention groups. 

**Table 1:** - Intervention group and pain scores, according to the PIPP scale. Fortaleza, Brazil, 2013

Intervention		Experimental Group 1			Experimental Group 2			Positive Control Group			Total			p-value*
		%	n		%			%	n		%	n		
Pain score														
	≤ 6	7	29.2		14	42.4		11	47.8		32	40.0		0.398
	≥ 7	17	70.8		19	57.6		12	52.2		48	60.0		

* Pearson's chi-square test

In Tables 2, 3, 4 and 5 presented below, neonatal variables are shown along with pain scores and allocation groups of the newborns studied.

**Table 2:** - Distribution of neonatal categorical variables of the PTI admitted to the Neonatal Unit, according to group allocation and the PIPP pain scores (T30) scale. Fortaleza, Brazil, 2013

Intervention	Variables		PIPP pain score*						Total		p-value^†^
			None/Minimum pain ≤ 6			Moderate/maximum pain ≥ 7					
			n	%		n	%		n	%	
Experimental Group 1	Sex										0,647
		Male	4	57,1		12	70,6		16	66,7	
		Female	3	42,9		5	29,4		8	33,3	
		Total	7	100		17	100		24	100	
	Type of birth										0,333*
		Normal	-	-		4	23,5		4	16,7	
		C-section	6	85,7		12	70,6		18	75	
		Forceps	1	14,3		1	5,9		2	8,3	
		Total	7	100		17	100		24	100	
Experimental Group 2	Sex										0,284
		Male	9	69,2		9	45		18	54,5	
		Female	4	30,8		11	55		15	45,5	
		Total	13	100		20	100		33	100	
	Type of birth										0,456*
		Normal	4	30,8		10	50		14	42,4	
		C-section	7	53,8		9	45		16	48,5	
Experimental Group 2		Forceps	2	15,4		1	5		3	9,1	0,456*
		Total	13	100		20	100		33	100	
Positive Control Group	Sex										0,389
		Male	8	72,7		7	58,3		15	65,2	
		Female	3	27,3		5	41,7		8	34,8	
		Total	11	100		12	100		23	100	
	Type of birth										1,000*
		Normal	2	18,2		3	25		5	21,7	
		C-section	8	72,7		9	75		17	73,9	
		Forceps	1	9,1		0	0		1	4,3	
		Total	11	100		12	100		23	100	

* Premature Infant Pain Profile

† Pearson's chi-square test or Fischer's Exact Test

**Table 3:** - Distribution of numerical neonatal variables of the PTI in EG1 (Music) and total PIPP values (T30). Fortaleza, Brazil, 2013

Variables	PIPP* Value	Mean±SD^†^	95% CI^‡^		Minimum	Maximum	p-value^¶^
			IL^§^	SL^||^			
Birth weight	≤6	2.380.6±581.4	2.115.9	2.645.2	1.336.0	3.054.0	0.077
	≥7	2.092.9±631.5	1.915.3	2.270.6	1.226.0	3.946.0	
	Total	2.176.8± 672	2.029.4	2.324.2	1.226.0	3.946.0	
Head circumference	≤6	32.8±2.1	31.8	33.8	29.5	35.0	0.003
	≥7	31.0±2.4	30.3	31.6	26.5	36.5	
	Total	31.5±2.4	30.9	32.1	26.5	36.5	
Chest circumference	≤6	29.4±3.1	28.0	30.9	23.0	32.0	0.032
	≥7	27.8±2.8	27.0	28.6	22.0	34.5	
	Total	28.3±3.0	27.5	29.0	22.0	34.5	
Number of punctures	≤6	1.0±0.0	1.0	1.0	1.0	1.0	0.080
	≥7	1.3±0.8	1.1	1.5	1.0	4.0	
	Total	1.2±0.6	1.1	1.4	1.0	4.0	
1’ Apgar	≤6	7.9±0.4	7.7	8.0	7.0	8.0	0.001
	≥7	6.2±2.2	5.6	6.8	2.0	9.0	
	Total	6.7±2.0	6.2	7.2	2.0	9.0	
5’ Apgar	≤6	8.7±0.5	8.5	8.9	8.0	9.0	0.047
	≥7	8.3±0.9	8.0	8.5	6.0	10.0	
	Total	8.4±0.8	8.2	8.6	6.0	10.0	
Corrected gestational age in weeks	≤6	35.2±1.4	34.5	35.8	32.6	36.3	0.003
	≥7	34.0±1.5	33.6	34.4	32.0	36.5	
	Total	34.4±1.5	34.0	34.7	32.0	36.5	
Corrected gestational age in days	≤6	247.1±9.2	243.0	251.3	230.0	255.0	0.008
	≥7	240.1±10.2	237.3	243.0	224.0	257.0	
	Total	242.2±10.3	239.7	244.6	224.0	257.0	
Cronological age in days	≤6	1.1±1.5	0.5	1.8	0.0	4.0	0.956
	≥7	1.2±2.6	0.4	1.9	0.0	9.0	
	Total	1.2±2.3	0.6	1.7	0.0	9.0	

* Premature Infant Pain Profile

‡ Confidence Interval

|| Superior Limit

† Standard Deviation

§ Inferior Limit

¶ Student's t-test or Mann-Whitney's U Test

**Table 4:** - Distribution of numerical neonatal variables of the PTI in EG2 (Music and 25% Glucose) and total PIPP values (T30). Fortaleza, Brazil, 2013

Variables	PIPP* Value	Mean±SD^†^	95% IC^‡^		Minimum	Maximum	p-value^¶^
			IL^§^	SL^||^			
Birth weight	≤6	2.247.8±638.5	2.043.6	2.452.0	1.352.0	3.620.0	0.499
	≥7	2.164.2±574.1	2.014.6	2.313.8	1.250.0	3.365.0	
Birth weight	Total	2.198.0±599.2	2.078.5	2.317.5	1.250.0	3.620.0	0.499
Head circumference	≤6	31.7±2.6	30.9	32.5	26.0	36.0	0.267
	≥7	31.2±2.2	30.6	31.7	26.5	35.0	
	Total	31.4±2.4	30.9	31.9	26.0	36.0	
Chest circumference	≤6	29.9±3.6	28.7	31.0	25.0	39.0	0.002
	≥7	27.8±2.7	27.1	28.5	23.0	34.0	
	Total	28.7±3.3	28.0	29.3	23.0	39.0	
Number of punctures	≤6	1.2±0.4	1.0	1.3	1.0	2.0	0.291
	≥7	1.3±0.5	1.1	1.4	1.0	3.0	
	Total	1.2±0.5	1.1	1.3	1.0	3.0	
1’ Apgar	≤6	5.7±2.6	4.9	6.5	1.0	9.0	0.009
	≥7	6.9±1.9	6.4	7.4	3.0	9.0	
	Total	6.4±2.3	6.0	6.9	1.0	9.0	
5’ Apgar	≤6	8.1±1.0	7.7	8.4	6.0	10.0	0.002
	≥7	8.6±0.8	8.4	8.8	6.0	10.0	
	Total	8.4±0.9	8.2	8.6	6.0	10.0	
Corrected gestational age in weeks	≤6	34.7±1.4	34.2	35.1	32.4	36.6	0.078
	≥7	34.2±1.4	33.8	34.5	32.3	36.6	
	Total	34.4±1.4	34.1	34.6	32.3	36.6	
Corrected gestational age in days	≤6	243.8±10.0	240.6	247.0	228.0	258.0	0.064
	≥7	240.1±9.7	237.5	242.6	227.0	258.0	
	Total	241.6±9.9	239.6	243.6	227.0	258.0	

* Premature Infant Pain Profile

‡ Confidence Interval

|| Superior Limit

† Standard Deviation

§ Inferior Limit

¶ Student's t-test or Mann-Whitney's U Test

**Table 5:** - Distribution of numerical neonatal variables of the PTI in PCG (25% Glucose) and total PIPP values (T30). Fortaleza, Brazil, 2013

Variables	PIPP* Value	Mean±SD^†^	95% IC^‡^		Minimum	Maximum	p-value^¶^
			IL^§^	SL^||^			
Birth weight	≤6	1.996.5±432.3	1.850.2	2.142.8	1.396.0	2.999.0	0.045
	≥7	1.769.2±491.3	1.595.0	1.943.4	918.0	2.578.0	
	Total	1.887.8±472.1	1.774.4	2.001.2	918.0	2.999.0	
Head circumference	≤6	30.6±2.0	29.9	31.3	27.5	35.5	0.027
	≥7	29.1±3.4	27.8	30.3	20.0	32.6	
	Total	29.9±2.9	29.2	30.5	20.0	35.5	
Chest circumference	≤6	28.2±2.3	27.4	28.9	24.0	32.0	0.073
	≥7	26.7±4.1	25.3	28.2	20.0	34.0	
	Total	27.5±3.3	26.7	28.3	20.0	34.0	
Number of punctures	≤6	1.1±0.3	1.0	1.2	1.0	2.0	0.231
	≥7	1.2±0.4	1.0	1.3	1.0	2.0	
	Total	1.1±0.3	1.0	1.2	1.0	2.0	
1’ Apgar	≤6	6.6±1.6	6.0	7.1	4.0	9.0	0.661
	≥7	6.4±2.5	5.5	7.2	2.0	9.0	
	Total	6.5±2.1	6.0	7.0	2.0	9.0	
5’ Apgar	≤6	8.3±0.7	8.0	8.5	7.0	9.0	0.564
	≥7	8.4±0.9	8.0	8.7	7.0	9.0	
	Total	8.3±0.8	8.1	8.5	7.0	9.0	
Corrected gestational age in weeks	≤6	33.6±1.6	33.0	34.1	32.0	36.6	0.421
	≥7	33.9±1.7	33.3	34.5	32.0	36.6	
	Total	33.7±1.7	33.3	34.1	32.0	36.6	
Corrected gestational age in days	≤6	235.8±11.8	231.9	239.8	224.0	258.0	0.478
	≥7	237.9±12.4	233.5	242.3	224.0	258.0	
	Total	236.8±12.0	233.9	239.7	224.0	258.0	
Cronological age in days	≤6	4.3±11.1	0.5	8.0	0.0	40.0	0.175
	≥7	1.5±2.3	0.7	2.3	0.0	8.0	
	Total	3.0±8.2	1.0	4.9	0.0	40.0	

* Premature Infant Pain Profile

‡ Confidence Interval

|| Superior Limit

† Standard Deviation

§ Inferior Limit

¶ Student's t-test or Mann-Whitney's U Test

Regarding the analysis of the therapeutic variables, type of oxygen therapy (mechanical ventilation, nasal CPAP, Oxi-Hood and room air), place of hospitalization (high risk, medium risk) and puncture site (left radial, right radial, left brachial or right brachial) according to the allocation groups and PIPP pain scores, the PTI were mainly concentrated around values ≥7, or moderate to maximum pain. The use of nasal CPAP as a method of oxygen therapy was present more often in pain scores ≥7. However, the PIPP scores were significantly different (p=0.012) according to the type of oxygen therapy used in PTI in EG2, with the Oxi-Hood frequently associated with scores ≤ 6 and CPAP with PIPP ≥7.

## Discussion

It is important to highlight that no experimental study had yet examined the pain reactivity in PTI or assessed the relationship between the neonatal and therapeutic variables and the non-pharmacological interventions of music, music plus 25% glucose, or 25% glucose, using a PIPP pain scale. This makes the current research unprecedented and innovative. 

Regarding the variables of sex and GA, a literature review that examined 18 articles from national and international databases on the effect of sex, GA and severity of the disease in neonatal pain reactivity in premature children. Fourteen studies evidenced that the IG variable interfered with the pain response of newborns. However, little evidence has been provided about the impact of sex on pain responses in those infants born extremely premature, especially at an early age of life^(^ 8 ^)^.

Specifically in relationship to sex, this study corroborates other authors^(^ 8 ^)^ regarding the lack of a statistically significant association for newborns allocated in EG1, EG2 and PCG, although it is shown that newborn males were more reactive to pain in the music group and the 25% glucose group. 

Supporting this result in relationship to music, a study that examined the differences in the responses of gender (masculine/feminine) to musical stimuli, subjects were exposed to 21 minutes of lullabies, alternating with three minutes of silence, by means of headphones. Responses obtained for physiological and behavioral parameters indicated no statistically significant changes for the studied variable; namely, neither the masculine nor feminine gender showed greater receptivity to listening to lullabies, nor did either show a tendency to present higher pain scores^(^ 9 ^)^.

Compared to the study proposed, when analyzing 60 newborns, 30 males and 30 females, GA ≥ 38 weeks, hospitalized at Holy House of Mercy Foundation of Pará, a study aimed to evaluate the expression of pain manifested by newborns submitted to two physiotherapy routine procedures, the thoracic manual vibrocompression and the manual diaphragmatic stimulation through validated scales (Neonatal Infant Pain Scale - NIPS and Neonatal Facial Coding System - NFCS), it was noted that newborn males had pain during thoracic manual vibrocompression. The females, on the other hand, showed no pain during physical therapy, using the NIPS or the NFCS assessments^(^ 10 ^)^.

An observational study was conducted in three neonatal intensive care units (NICU) in Canada, with a sample of 149 PTI and full-term newborns, comparing the physiological and behavioral pain responses of infants at risk during the neonatal period and at six months of age in different GA. Using the painful procedure of heel-lancing, newborns at GA <27 weeks were found to have pain responses that were similar to those of newborns at higher GA (28-31 weeks, 32-35 weeks and > 36 weeks^(^ 11 ^)^, which resembles the newborns allocated in EG2 and PCG, whose GA did not influence the pain response in PTI.

Another study evaluated the sensitivity and specificity of two behavioral scales (NFCS and NIPS) in newborns at different GA, with 113 newborns, 5 ' Apgar score >7, divided into three groups - 28-33 weeks (group A), 34-37 weeks (group B) and 38-41 weeks (group C) - during lancing (L) and friction (F) procedures in groups A-P (n=17, 1.5+0.4kg procedure); A-F (n=18, 1.5+0.4kg); P-B (n=25, 2.5+0.5kg); B-F (n=25, 2.4+0.6kg); C-P (n=23, 3.3kg+0.5kg); C-F (n=25, 3.3+0.4kg). When comparing the groups (Groups A-P, B-P and C-P), no statistically significant differences among the three groups were observed throughout the study period, either with the NFCS or the NIPS scales (K. Wallis: p> 0.05)^(^ 12 ^)^.

A comparative quasi-experimental study performed at the Maternity Unit in the interior of São Paulo state evaluated 40 newborns at term during the vaccination procedure against Hepatitis B through the NFCS. The results identified a significant association between groups regarding GA in days (p = 0.02), however, it was not clinically relevant, since the two groups were comprised of term infants. Furthermore, it was identified that the mean birth weight of newborns, 3.190g for those immediately placed in skin to skin contact with the mother after delivery, and 3.325g for those taken directly to the heated crib, showed no association between the groups (p=0.29)^(^ 13 ^)^. In the present study on the other hand, weight showed a statistically significant difference for the premature in PCG (p=0.045). 

Regarding the Apgar investigation, in the experimental group (EG1 and EG2), the PTI was statistically significant for 1' and 5' Apgar, although the mean 5' Apgar score was above seven.

A clinical trial conducted in eastern Canada aimed to evaluate the effect of co-sleeping in recovery and response to pain, using the PIPP with 67 premature twin neonates at GA between 28 and 36 weeks, divided into two groups: those treated in the same incubator or crib (co-sleeping) and another group that received care in separate incubators or cribs. Comparing the groups, there was a statistically significant difference on the day of heel lance regarding the 5 'Apgar variable (p=0.05^(^ 14 ^)^.

In the investigation of Apgar and the number of punctures, a study aimed at assessing the behavioral and physiological responses of PTI undergoing heel lance divided neonates into two groups. The first, with a mean gestational age of 27.3 weeks, and corrected GA 32-32 6/7 weeks; the second, at 32.3 weeks, observed on the 4th day of life. There was no association between the number of painful procedures with a higher HR or lower SpO_2_. The facial expressions of pain were less evident in those subjected to a greater number of painful procedures, and the highest Apgar values ​​ significantly contributed to raising the scores related to facial expressions^(^ 15 ^)^.

Regarding the evolution of head circumference, another investigation with 63 PTI at GA between 28 and 33 weeks, who heard classical music (Mozart) for 20 minutes on two consecutive days, found no statistically significant difference among hospitalized newborns in the NICU undergoing music therapy and the control group^(^ 16 ^)^.

Regarding therapeutic variables, a quantitative longitudinal before-and-after study in a NICU in Fortaleza, CE, investigated the physiological parameters of RR, HR, pulse rate (P), SpO_2_, before, immediately after and five minutes after performing tracheal (TA) and upper airway aspiration (UAA) of 104 newborns. Results showed that newborns using an Oxi-Hood suffered the biggest changes in respiratory function, manifested as trouble returning to pre-aspiration RR values​​, differing from the results of the present study^(^ 17 ^)^.

An experimental study with 20 PTI undergoing installation and reinstallation of nasal CPAP indicated that when this artifact was installed, 100% of newborns felt pain. However, when they were offered non-nutritive sucking (minimum of a gloved finger in the mouth of the NB), the premature infants reacted to the stimulus without reaching an indicative pain score through NIPS^(^ 18 ^)^, similar to the EG1 and PCG.

## Conclusion

The study design was adequate to the proposed objectives. When analyzing the relationship of neonatal and treatment variables with PTI undergoing arterial puncture for blood collection while exposed to either music, music and 25% glucose, or 25% glucose alone, it was found that the three study groups were heterogeneous in some variables. This is an important aspect because some of these variables showed significant differences regarding pain patterns: 1' Apgar, 5' Apgar, gestational age, weight, HC, CC and type of oxygen therapy with p <0.05.

Among the study's limitations is the inclusion of PTI on oxygen therapy, because they had PIPP scale scores significantly higher than the others, which indicates that such a situation should perhaps have been considered as an exclusion criterion. 

The contribution is the encouragement of performing a clinical trial with PTI undergoing arterial puncture under three types of interventions for pain relief: music, music and 25% glucose and 25% glucose, with pain being assessed through the multidimensional PIPP scale, and its relationship with neonatal and therapeutic variables in the NICU.
